# Tests of Uniaxial Compression of Single Grains

**DOI:** 10.3390/ma17225479

**Published:** 2024-11-09

**Authors:** Iwona Radosz, Magdalena Pietrzak, Leszek M. Kaczmarek

**Affiliations:** Faculty of Civil Engineering, Environmental and Geodetic Sciences, Koszalin University of Technology, Śniadeckich 2, 75-453 Koszalin, Poland; magdalena.pietrzak@tu.koszalin.pl (M.P.);

**Keywords:** uniaxial compression, granular material, crushing force, single grain, quartz sand, glass granules, crushed glass grains

## Abstract

Tests of the uniaxial compression of single grains were performed in a specially designed press, which allowed the recording of an applied load in regard to the time and observation of occurring phenomena in a polarization assay. Three types of grains were tested: quartz sand, glass granules, and crushed glass. The strength tests showed different mechanisms of grain damage depending on the type of grain. In addition, the formation and spread of interference fringes, forming “chains of force” in samples with a large number of grains, were observed by testing glass grains under the polarization assay. A more detailed understanding of the strength characteristics of single grains will allow the verification of the models most commonly used in DEM.

## 1. Introduction

In material science, assessing the mechanical properties of materials, particularly compressive strength, is essential for the performance and safety in engineering applications. Uniaxial compression testing is a key method for understanding the compressive strength and elastic modulus of homogeneous materials like metals and ceramics [1,2]. For heterogeneous materials, such as composites or rocks, this testing accounts for the internal nonuniformities that affect the failure mechanisms [3,4]. The point load test is useful in field conditions for rock strength evaluation, showing the correlations with uniaxial compressive strength [1]. Other methods, like penetration tests in geotechnics, assess soil and rock hardness, revealing their compressive properties [5]. For brittle materials, impact tests capture the energy absorbed during fracture [6]. Advanced techniques, such as micro- and nanoindentation, provide detailed insights into the mechanical properties at smaller scales, improving the understanding of the compressive behavior across various materials [7]. These approaches collectively enhance the material characterization and advance the research and practical applications in materials engineering.

At the end of the 20th century, in soil mechanics, attention was drawn to the influence of single grain strength on the deformation characteristics of granular materials. Many research centers began to investigate the micro- and macro-scale relations in order to better relate the mathematical models to the strength characteristics of single grains.

One commonly used test, in soil grain experiments, became uniaxial compression tests. In 1998, McDowell and Bolton [8], using such tests, demonstrated that the stresses in soil are determined by the tensile strength of the individual grains. The results of similar experiments by Nakata’s team [9,10] in 2001 showed that the size, roundness, mineralogy, and compressive strength of single grains affect the behavior of soil in macroscopic tests. They also defined five stages of grain damage, before and after edometric testing.

Cavarrett and O’Sullivan [11,12] confirmed the five-stage model of loaded grain behavior and showed that actual grain loading is preceded by roughness crushing and/or grain rotation during the initial loading phase. However, Russell and Muir Wood’s [13] study showed that due to the rapid brittle cracking and complex internal structure of sand particles, the damage mechanisms of soil grains are still not well understood. Moreover, it is unclear whether several existing cracking mechanisms (proposed for ideal particles) actually correspond to those occurring in natural sand grains.

To investigate the cracking of quartz sand grains, Zhao et al. [14] conducted grain compression tests using microscopic X-ray tomography (CT). The study focused more on the characterization of the fragments after grain damage rather than the dynamic cracking process (this was due to the limitations of the CT method). In addition, Wang and Coop [15] continued similar experiments with CT, using a high-speed microscopic camera. Although the observation of the cracking process was limited to the external view, they were able to monitor the damage initiation and progress. Based on the visualization of the destruction process and the number of fragments formed after damage, they distinguished four types: division, explosion, crushing, and mixed type. They concluded that the more round grains tend to have explosive cracking at a higher damaging force, and the less round grains tend to split into smaller pieces. Additionally, research using an advanced method of X-ray CT imaging continues to provide valuable information on how the individual grain characteristics, such as the morphology and mineral composition, affect the overall soil behavior. In one study, single grain crushing tests under railway loading conditions demonstrated how the fragmentation of sand particles influences the adhesion coefficients in rail contacts [16]. This emphasizes the growing interest in how grain characteristics affect real-world applications.

A lot of single grain studies have been conducted to improve the quality of the DEM modeling of granular media. In the last few years, there have been published papers on studying the effect of sphericity, circularity, and the roughness coefficient [17,18] and papers on Weibull distributions—commonly used in the statistical calculations of granular materials [19,20]. Recent studies have advanced the understanding of this phenomenon. For instance, a 2023 study explored the breakage of coarse sand particles under direct shear tests, utilizing 3D DEM simulations. This research highlighted that crushable particles show significant changes in their stress–strain relationships, leading to a transition from dilative to contractive behavior, and the accumulation of finer particles post-crushing. These insights are crucial for modeling granular materials in geotechnical applications [21]. Another 2023 study applied the Discrete Element Method (DEM) to examine the behavior of crushable coarse sand under direct shear conditions. The research highlighted that particle breakage leads to an accumulation of finer particles, which subsequently alters the stress–strain relationships and promotes contractive rather than dilative behavior. This shift in behavior has important implications for the stability of granular structures like earth dams [21].

Recent advances also include the use of mesoscale computational simulations, which integrate the effects of grain shape and distribution, allowing for more accurate predictions of soil–rock mixtures under a load. Such simulations help in understanding the degradation processes and particle breakage that are vital for applications like slope stability and earth dam construction [22].

Incorporating these findings into DEM models is crucial for improving the accuracy of the simulations used in engineering practice, especially for materials where particle breakage plays a significant role. This research directly complements the earlier works by Bolton, McDowell, and Nakata, enriching the existing models with a deeper understanding of the particle fragmentation dynamics.

## 2. Materials and Methods

### 2.1. Compressive Strength Testing of Grains

Uniaxial compression tests were conducted in a specially designed miniature press. The press has a force measurement range of up to 10 kN and allows photographic observation under white light and a polarization assay. The grains were loaded in a quasi-static manner until damage. The program integrated into the mini press automatically recorded ten force value measurements per second, the value of the maximum compressive force and a “load–time” graph.

Uniaxial compression tests were conducted individually on a total of 250 single glass grains, comprising 100 non-thermally toughened glass granules, 100 thermally toughened glass granules, and 50 crushed glass crumbs. Each single glass grain had a diameter ranging from 0.8 mm to 1.1 mm.

**Figure 1:**
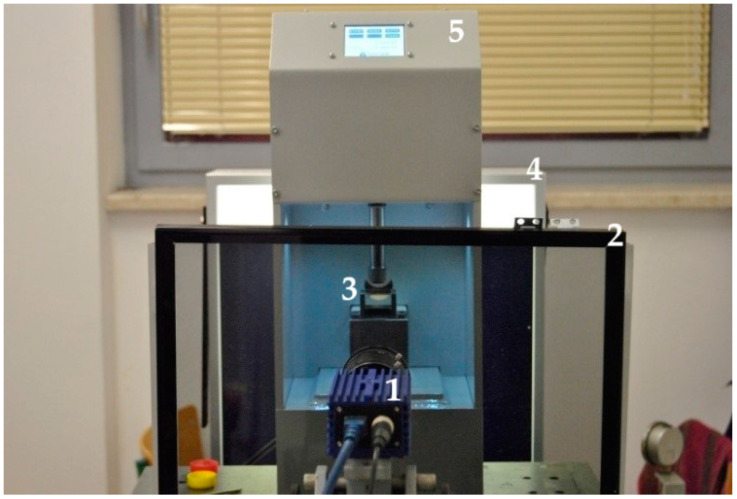
View of the measurement station (mini-press in the polarizer optical system): 1—digital camera, 2—analyzer, 3—tested model, 4—polarizer, 5—miniature press.

Some of the glass grain tests were recorded under the circularly polarization assay using a digital camera. In elastooptical tests, a large-dimensional polariscope was used, where the analyzer and the quarter-wave are integrated, while the polarizer is integrated with the quarter-wave and permanently connected to the light source (white or monochromatic light source of a 565 nm wavelength) (Figure 1).

### 2.2. Eastooptical Testing

Elastooptical tests can significantly help in the identification of stress and strain fields. Their main advantage is the ability to determine the stress state inside the model under study and in real objects, provided the scale of similarity is taken into account. Elastooptics has been used in the study of granular materials since the second half of the 20th century. Earlier it was used to study transparent, amorphous, normally isotropic solid materials [23].

**Figure 2:**
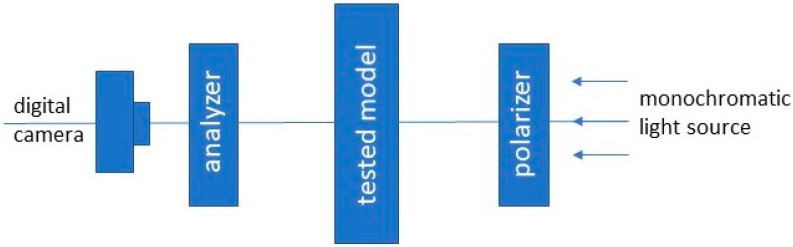
The optical system of transmission polarizer.

A polariscope is an optical instrument in which a light source passes through a polarizer and then through a sample and an analyzer (Figure 2).

A necessary condition in elastooptical testing is the so-called transparency of the tested material. Every transparent material, although made of transparent grains like glass, in its mass is an opaque medium. This is a result of the light scattering caused by its reflection on the walls of individual grains. In order to make the medium completely transparent, all pores (spaces between grains) should be filled with a colorless liquid with the same refractive index as glass, i.e., an immersion liquid [23].

Model studies on the non-cohesive elastooptic materials for soil mechanics problems have a relatively long history. Such studies on model bulk media were carried out by, among others: Dantu [24], Wakabayashi [25], Drescher [26], Allersma [27] and Dyer [28]. By observing the photographs taken under the polarization assay, they were able to identify characteristic bright bands representing loaded chains of grains. It was concluded that these bands coincide with the principal stress trajectories.

In recent years, due to the emergence of new tools (i.e., digital photography, PIV image analysis, DEM simulation), there has been a return to this method of making observations.

**Figure 3:**
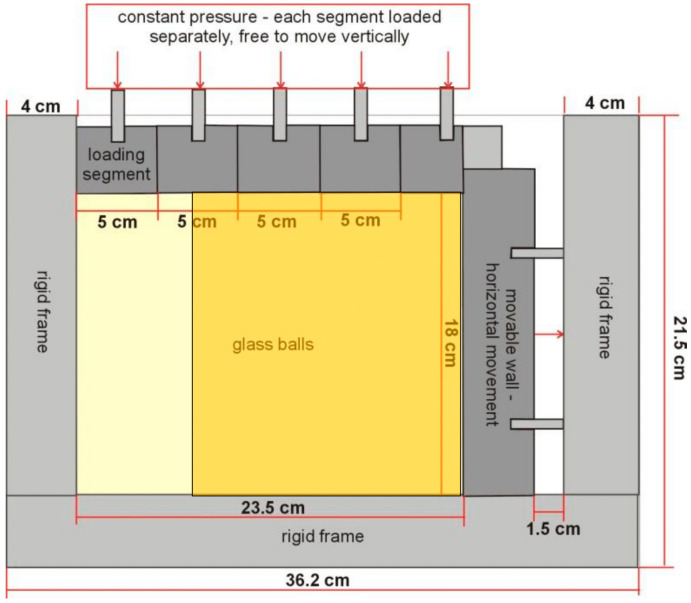
Experimental setup of the retaining wall model with the analysis area marked [33].

From 2009 to 2015, Lesniewska and Muir Wood carried out an elastooptical study of the problem of pressure on retaining walls [29,30,31,32]. The experiments were continued at the Koszalin University of Technology, where a model of a loaded retaining wall was also analyzed, with the primary aim of the precise identification of the evolution of the strain field in a granular medium. The experimental setup was described in detail in [29,30,31,32]. Small-scale tests on granular samples retained by using a movable rigid wall were performed in a glass-sided box (Figure 3). The glass sides were 20 mm thick and loaded under the lateral pressures from the granular material. The particular configuration included a smooth and rigid vertical wall, 180 mm high, supported by rods that were able to slide horizontally through the box. An active earth pressure mode was investigated, where a retaining wall moved away from the backfill. The mode of the test was quasi static with a constant wall displacement increment equal to 0.0625 mm (1/20 of the supporting screw lead). The tests were recorded by using a common digital camera, the Sony Cyber shot, with the resolution of 2560 × 1920 pixels. One average grain was represented by ~10 × 10 pixels. Photographs were taken at each wall displacement step. Experimental displacement fields were determined on their base, and hence, the strains were calculated using 2D digital image correlation (particle image velocimetry) DIC (PIV). Starlitbeads1000 spherical glass granules were used to form a granular sample ($d_{50}$ = 1.1 mm). Only dense samples were investigated. The glass granules were selected to represent soil due to their transparency. The tests were registered both in ordinary and polarized light to give not only strain but also stress information based on the photo-elasticity. Due to the demands of the photo-elastic method, the granular specimen was saturated with clove oil, having the same refraction index as glass [29,30,31,32].

**Figure 4:**
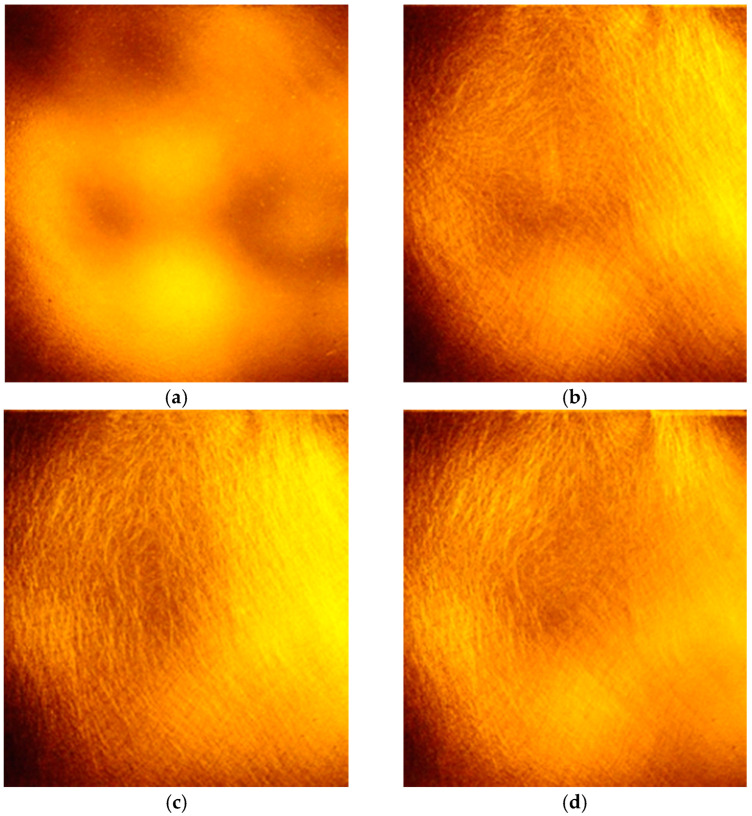

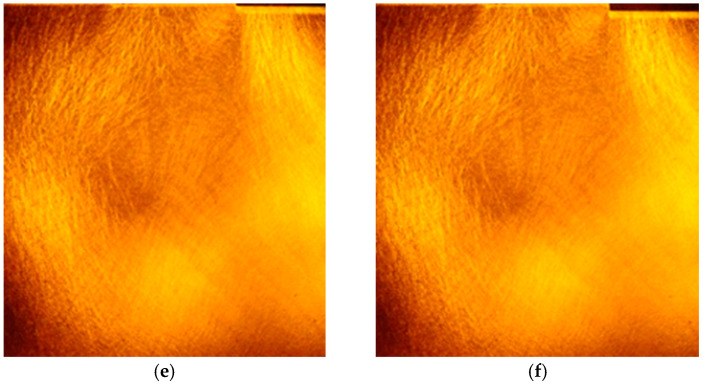
Fragment of a model study of the retaining wall, recorded in circularly polarization assay, p—load level [MPa] and d—total displacement [mm] [33]: (**a**) p=0.00 MPa, d=0.00 mm; (**b**) p=1.60 MPa, d=0.00 mm; (**c**) p=3.20 MPa, d=0.00 mm; (**d**) p=3.20 MPa, d=0.50 mm; (**e**) p=3.20 MPa, d=1.25 mm; (**f**) p=3.20 MPa, d=2.10 mm.

Figure 4 shows photos of the loaded model of this wall taken under the polarization assay. The study area is marked in Figure 3. A clear structure formed by “chains of loaded grains” can be seen emerging as the load increases [33].

**Figure 5:**
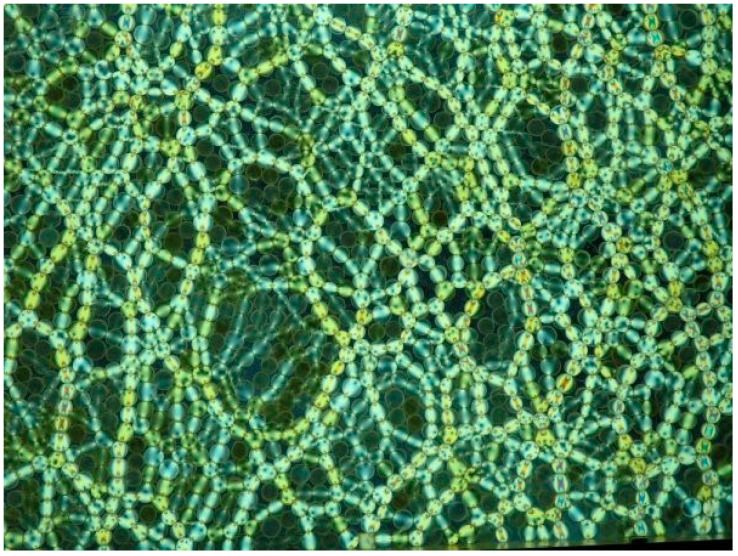
Force chains in a fabric of photoelastic discs [34].

During the experiments on the granular samples and the observation of “force chains”, it was noticed that the elastooptical effect in the form of light and dark interference fringes occurred individually in each sufficiently loaded grain (Figure 5). This gave rise to further studies aimed at determining the nature of the phenomena occurring at the grain scale [33,34,35].

### 2.3. Tested Materials

**Figure 6:**
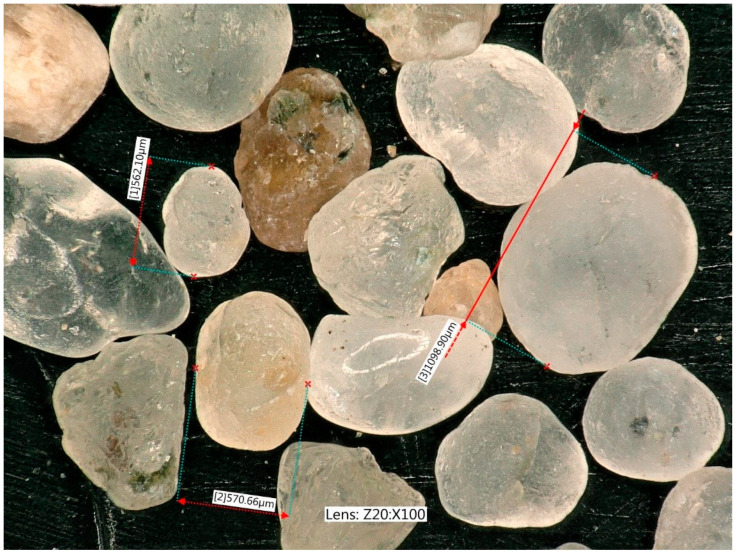
Quartz sand grains, magnified 50 times.

The following materials were tested: quartz sand—diameter d50 ~1.1 mm. Sand grains are irregular, mostly convex, but with small concavities (Figure 6) [36,37].

**Figure 7:**
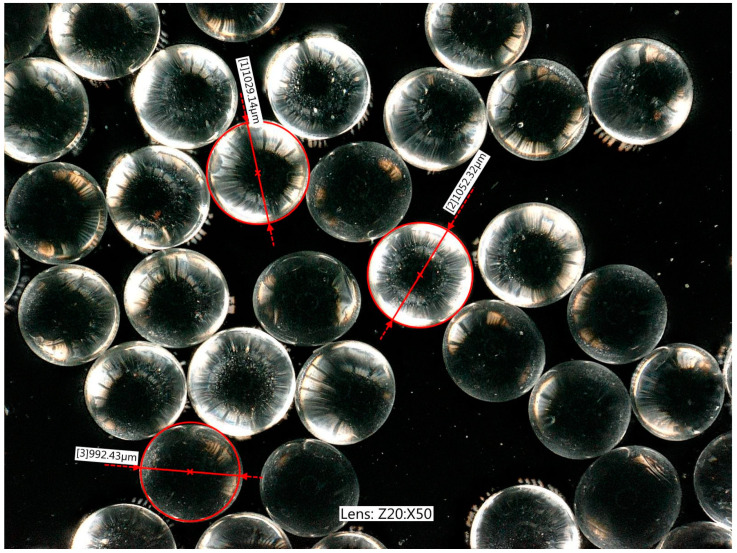
The largest fraction of Starlitbeads1000 (glass granules), magnified 50 times.

Starlitbeads1000 glass granules—diameter d50 ~1.1 mm. Figure 7 shows the glass granules represented by the fraction 1.0 mm (the largest grain diameter was 1.4 mm), magnified 50 times. Four fractions were tested in the compression tests: 0.8, 1.0, 1.2, and 1.4 mm. The granules were not perfectly spherical, but had a regular convex shape. Their additional characteristic was that the granules in their natural state have some initial stress due to rapid cooling during the manufacturing process. In the uniaxial compression tests, the granules in their natural state (non-toughened and without pressure) and grains after toughening were examined [36,37].

**Figure 8:**
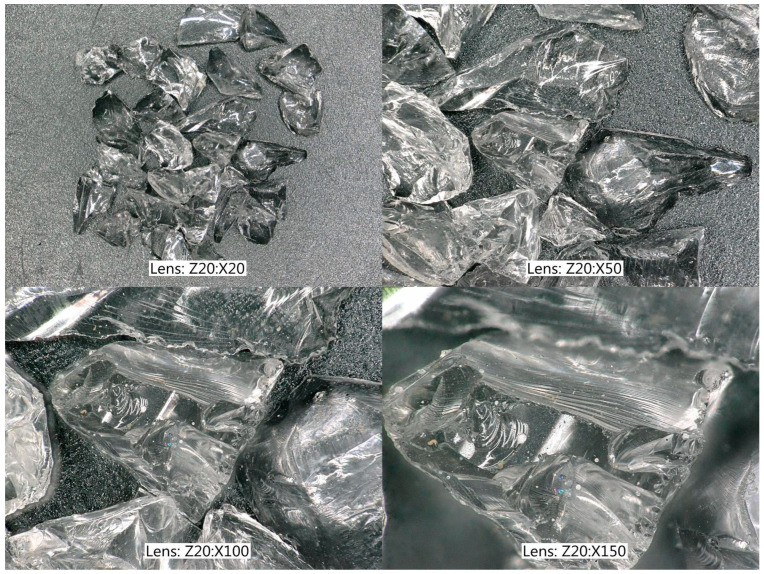
Crushed glass grains, magnified 20, 50, 100, and 150 times (Pyrex glass).

Crushed glass grains were formed by crushing a Pyrex glass sheet, resulting in granules with very irregular shapes and sharp edges (Figure 8), but without internal initial stresses.

According to the procedure adopted for the elastooptical testing of the granular materials made of glass (with a refractive index of 1.54), the condition necessary to observe the elastooptical effect, i.e., the transparency of the tested material, must be met. An immersion liquid, clove oil, with a similar refractive index of 1.533, was used in this study [36,37].

## 3. Results and Discussion

**Figure 9:**
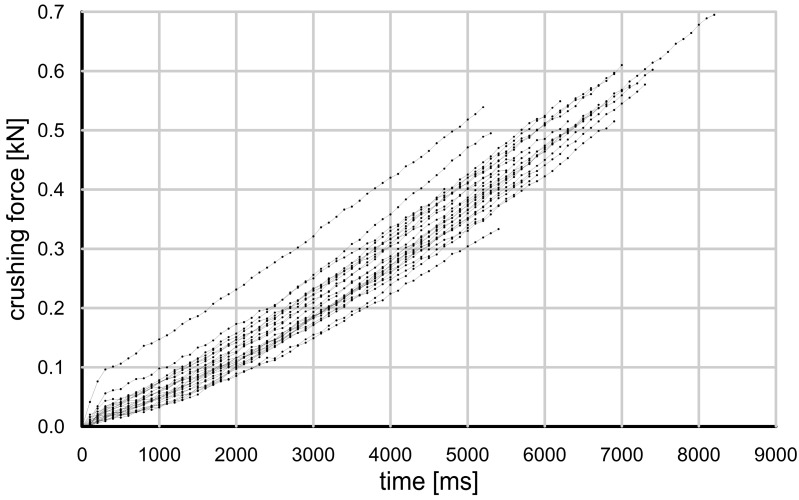
Grain crushing test results—crushing force value as a function of time for glass granules.

**Figure 10:**
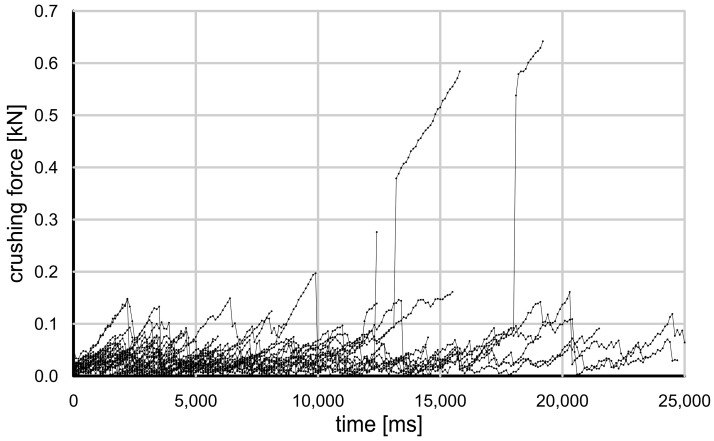
Grain crushing test results—crushing force value as a function of time for crushed glass grains.

In the strength tests performed, the grain damage mechanisms were different for each type of grain tested. Figure 9 and Figure 10 show the evolution of the crushing force for the glass granules and crushed glass grains.

It can be seen that the glass granules were damaged at a much higher force than the crushed glass. The change in force over time was monotonic and nearly linear. At some point, the glass granules suddenly disintegrated, and the remnants were so fine that they could not bear any additional load.

The crushed glass grains showed a completely different behavior—they split into relatively large pieces at a much lower loading force, and each piece retained some of its strength. As a result, the compressive force varied nonlinearly and it was difficult to determine the actual moment of grain damage. This behavior is typical of brittle cracking.

When the glass granules reached their maximum strength, they underwent what Wang and Coop [15] describe as an “explosion”, resulting in very fine fragments that did not carry even a fraction of the load. The crushed glass grains, when loaded, also disintegrated, but into much larger pieces—they were resistant to further damage and able to carry some load (“splitting” according to Wang and Coop) [15].

**Figure 11:**
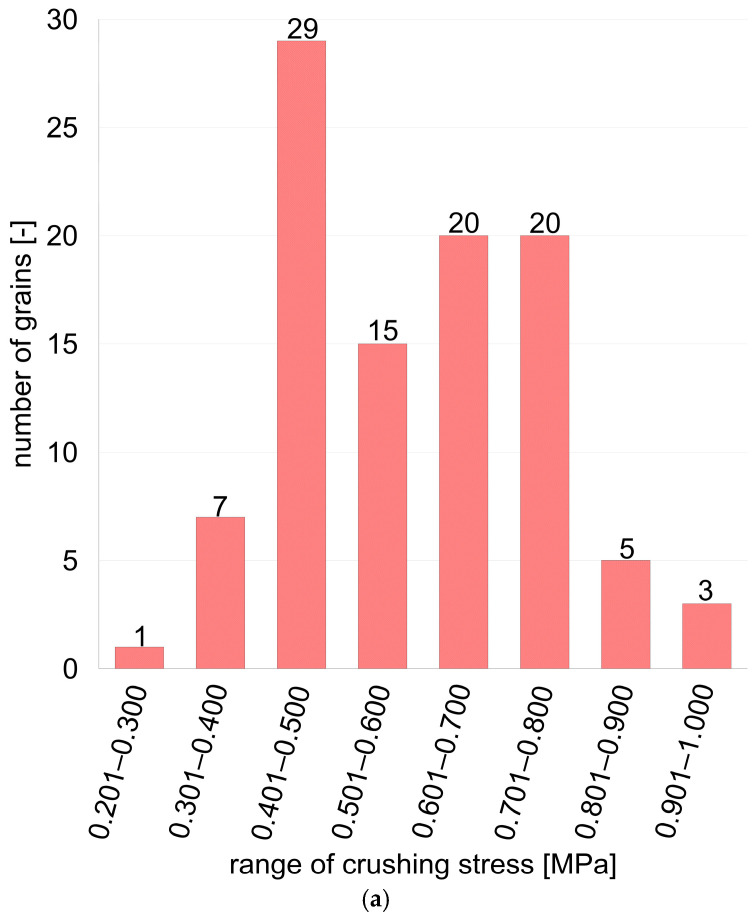

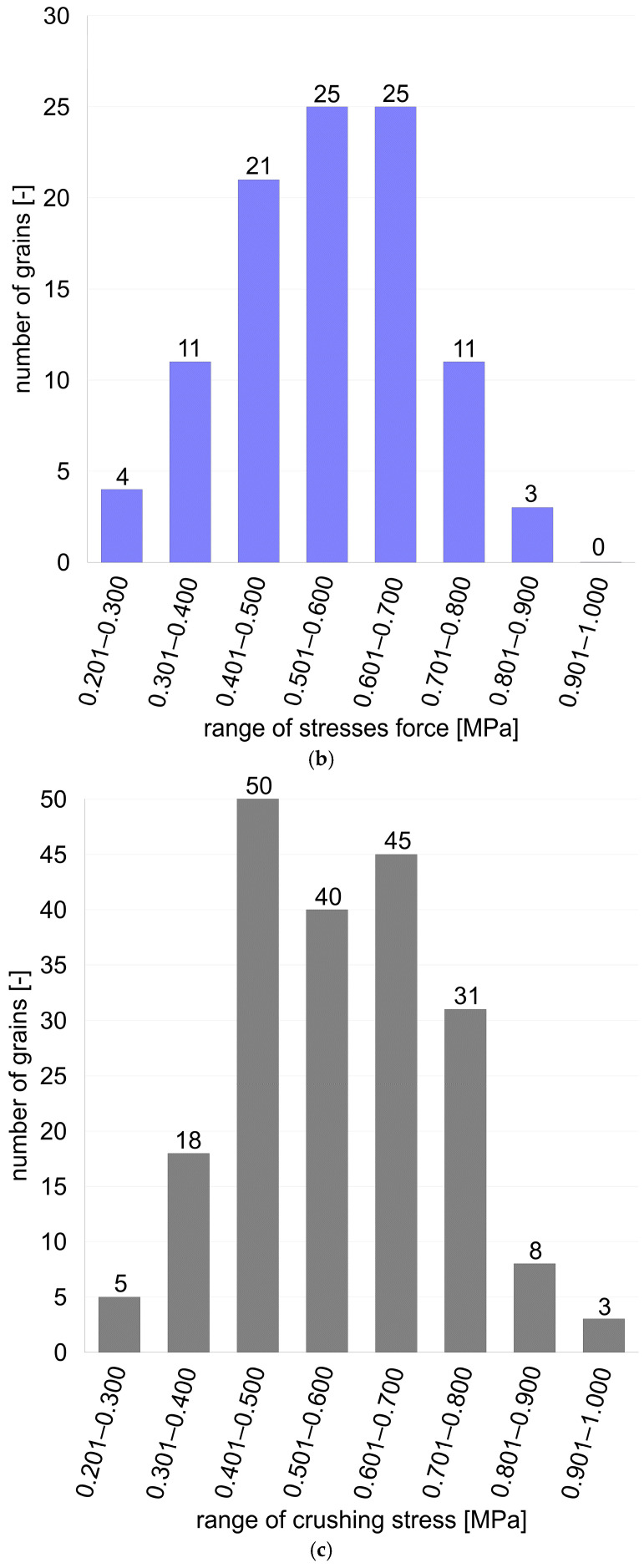
(**a**) Distribution of crushing stress for: (**a**) 100 grains sample for raw granules; (**b**) 100 grains sample for tempered granules; (**c**) 200 grains sample for grand total granules.

The statistical distribution of the grain crushing force shows the classical character of a Gaussian distribution (Figure 11). In the case of the raw granulates (Figure 11a), the distribution of crushing force shows a clear maximum in the range of 0.401–0.500 MPa, where the largest number of grains (29) was recorded.

In the case of the hardened granulates (Figure 11b), the distribution of the crushing force slightly shifted towards higher values. The largest number of grains (25) was recorded in the range of 0.501–0.600 MPa and 0.601–0.700 MPa.

The total distribution for 100 grains together, taking into account both raw and hardened granulates (Figure 11c), shows that the largest number of grains (50) was in the range of 0.401–0.500 MPa, and another 45 in the range of 0.601–0.700 MPa. This distribution indicates that most of the tested granulates had a crushing force in the range of medium values (0.401–0.700 MPa). It is worth noting the relatively low share of grains with a very high crushing force (0.801–1.000 MPa).

The analysis of the crushing force distribution of the glass granules revealed the significant differences in strength between the raw and tempered samples. As shown in Figure 11b, the tempered granules exhibited a shift in the peak of the distribution towards higher crushing forces (0.501–0.700 MPa). Despite this shift, it was observed that the number of granules with the highest strength (above 0.800 MPa) significantly decreased after tempering, suggesting that the tempered granules were more susceptible to failure compared to the raw granules. Additionally, in contrast to the raw samples, which displayed a more dispersed force distribution, the tempered samples had a narrower strength range, indicating a reduced resistance to varied loads. Thus, despite their increased uniformity, the tempered granules exhibited a lower overall crushing strength across a wider range of forces.

**Figure 12:**
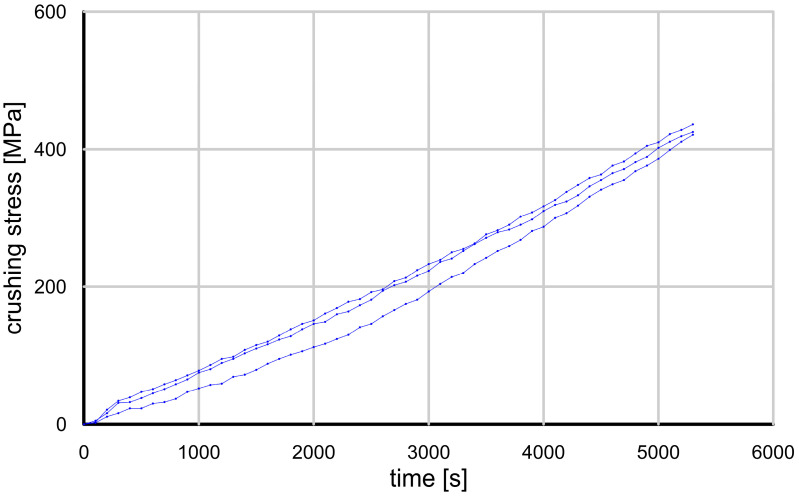
Results of grain crushing tests conducted on selected grains of glass granules for the full stress range (zero to ultimate strength).

**Figure 13:**
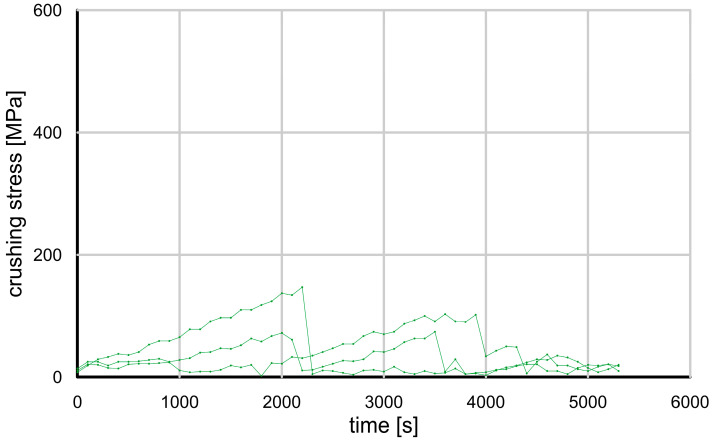
Results of grain crushing tests conducted on selected crushed glass grains for the full stress range (zero to ultimate strength).

**Figure 14:**
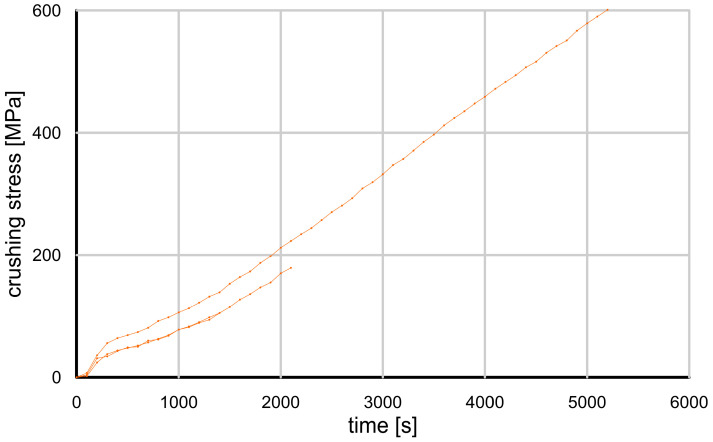
Results of grain crushing tests conducted on selected grains of sand for the full stress range (zero to ultimate strength).

To further investigate the different grain behavior, the dependence on grain size must be eliminated. This can be achieved by estimating the stress acting on a single grain (Figure 12, Figure 13 and Figure 14). The preliminary results show that while the strength of the sand and crushed glass grains was similar, the stress curve during loading was different.

If comparing the stress curves of the granules and sand grains, it is found that the curves are approximately linear, and at lower stress values (common in geotechnical practice or the physical modeling of geotechnical problems), the difference in the loading properties between the granules and sand is less significant.

**Figure 15:**
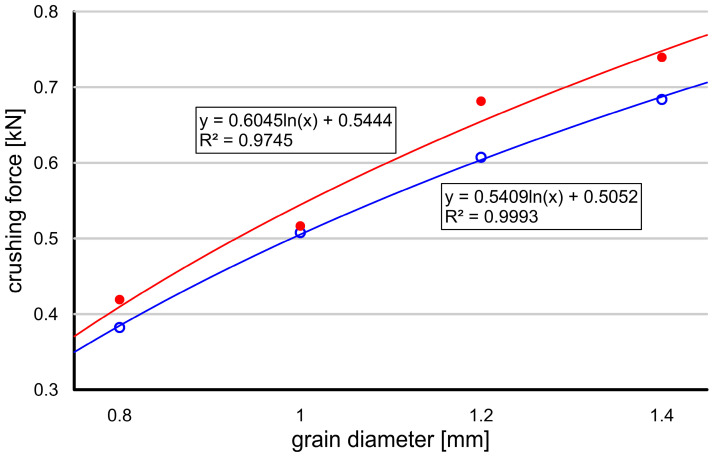
Results of grain crushing tests conducted on selected grains of sand for the full stress range (zero to ultimate strength).

Figure 15 illustrates the relationship between the average crushing force and grain diameter, with each diameter represented by 50 tests. The crushing force shows a nonlinear increase with the change in grain size, as indicated by the logarithmic trend fitted to the data, presented by the two curves with different coefficients. This behavior deviates from that observed in natural soil grains, which typically exhibit a reduced strength as the grain size increases. The discrepancy can be attributed to the presence of internal cracks, which are more likely in natural grains and contribute to their lower strength. The fitted logarithmic models (with the equations shown on the graph) suggest a strong correlation between the grain diameter and crushing force, as reflected by the high $R^{2}$ values, indicating a good fit to the experimental data.

**Figure 16:**
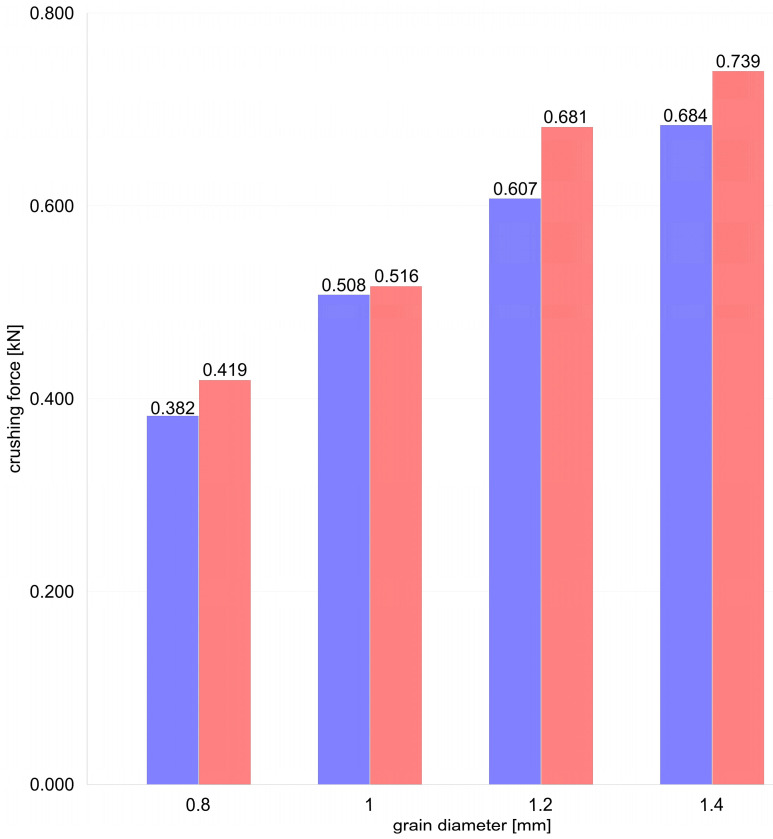
The relationship between crushing force and the grain diameter is presented in the form of a bar graph (red—raw grains, blue—thermally tempered grains).

Figure 16 shows the dependence of the crushing force on the grain diameter, comparing the raw grains (red) and heat-treated grains (blue). In each case, the raw grains show a slightly higher resistance to crushing than the heat-treated grains. For the grains with a diameter of 0.8 mm, the crushing force is 0.419 kN for the raw grains, while for the heat-treated grains, it is 0.382 kN. With an increase in the grain diameter to 1.0 mm, these values increase to 0.516 kN for the raw grains and 0.508 kN for the heat-treated grains, respectively. For a diameter of 1.2 mm, the crushing forces are also higher for the raw grains (0.684 kN) compared to the processed grains (0.607 kN). Finally, for the largest grain diameter, i.e., 1.4 mm, the crushing force is 0.739 kN for the raw grains and 0.681 kN for the heat-treated grains. The analysis shows that the raw grains always have a higher crushing strength than the heat-treated grains, regardless of the diameter. The heat treatment seems to reduce the crushing strength of the grains, which is particularly noticeable at larger diameters.

**Figure 17:**
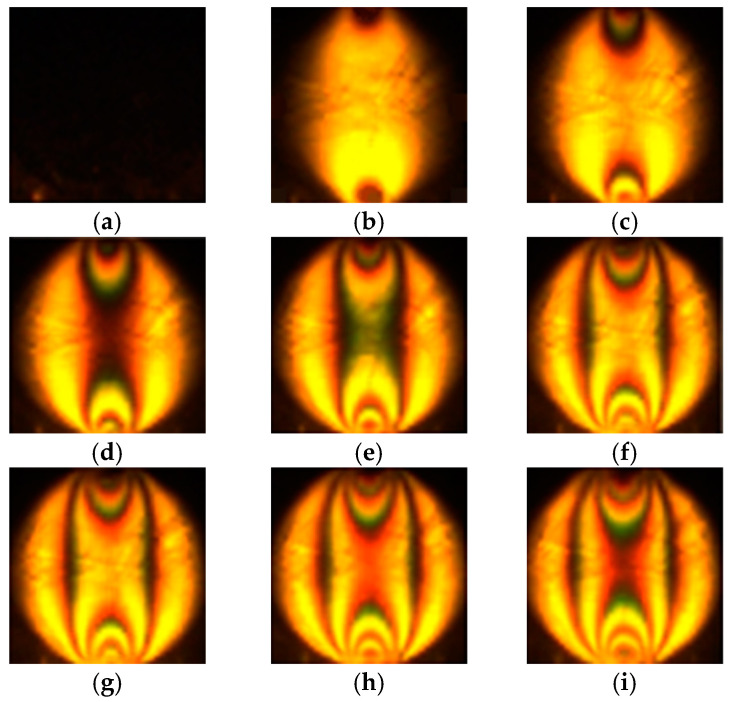

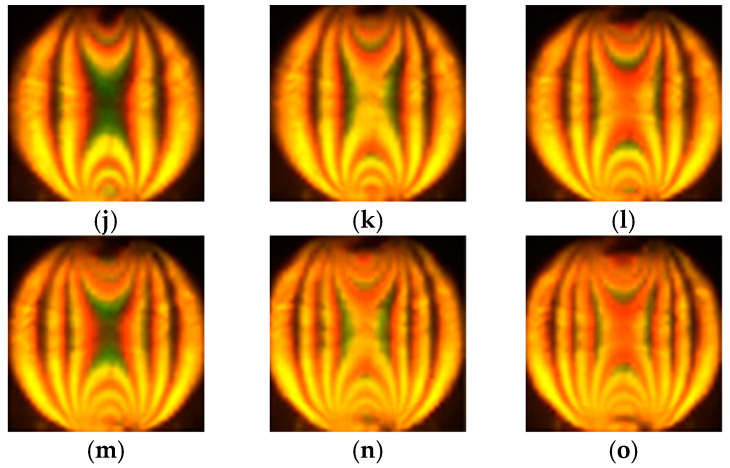
Compression of a single glass granule (diameter ~1 mm) loaded in the vertical direction, observed under circularly polarization assay, in regard to time: every half second, for the compressive force: (**a**) 0 [kN], (**b**) 0.888 [kN], (**c**) 1.603 [kN], (**d**) 2.108 [kN], (**e**) 2.778 [kN], (**f**) 3.105 [kN], (**g**) 3.536 [kN], (**h**) 3.898 [kN], (**i**) 4.281 [kN], (**j**) 4.776 [kN], (**k**) 5.443 [kN], (**l**) 6.167 [kN], (**m**) 7.056 [kN], (**n**) 8.014 [kN], (**o**) 8.604 [kN].

Single-grain tests are useful for interpreting larger-scale tests. The photographs taken during one of the tests under the polarization assay are illustrated in Figure 17. The formation and spread of the interference fringes can be seen.

It is evident from the images in Figure 17 that as the load increases, not only does the intensity of the image change, but the extent of the area of increased intensity also varies. In Figure 17b, the area of the increased intensity is relatively narrow, occupying about half the volume of the grain, while in Figure 17o, it covers almost the entire volume of the grain. This indicates that both the high intensity of the image and the area in which it occurs provide valuable information about the stress level. This assumption justifies a similar approach to “force chains” as represented by DEM users—typically, the width of an area is proportional to the value of the normal force acting on the grain.

## 4. Conclusions

The uniaxial compression tests of single grains revealed distinct damage mechanisms depending on the type of grain. The glass granules exhibited a higher resistance to damage compared to the crushed glass, with the former displaying a linear increase in the compressive force before sudden failure, while the latter fractured into larger pieces and retained some load-bearing capacity post-failure.

The tests indicated that hardening did not improve the mechanical strength of the glass granules. There was a slight shift in the distribution of the crushing force towards higher values. The process may not have a substantial impact on enhancing the mechanical properties of the tested materials.

The study found a nonlinear relationship between the crushing force and grain size, which deviates from the expected behavior in natural soil grains due to the internal cracks in natural materials. The raw grains consistently displayed a higher crushing strength compared to the heat-treated grains, across all the grain diameters.

The elastooptical tests confirmed the spread of the interference fringes during the compression, indicating the stress distribution within the grains. The extent of the stressed area increased with the load, allowing for a better understanding of the force chains in granular materials.

The results suggest that within the range of practical stresses, glass granules substitute quartz sand well —both have similar monotonic stress–deformation characteristics in a similar stress and deformation range. Glass granules are better suited for analyzing the load transfer in granular materials, while crushed glass is more suitable for modeling densification associated with grain crushing.

The test results presented in the article provide valuable insights into the behavior of granular materials under compressive loads, which has practical applications in soil mechanics, especially in model studies such as retaining walls, foundation stability, and slope behavior. Glass granules, as tested in the study, offer a useful substitute for natural sand due to their similar deformation properties under comparable loads. The uniformity, transparency, and predictable behavior of glass granules make them particularly advantageous for model studies where the detailed observation of stress and strain distribution is required.

In soil mechanics, understanding the stress–deformation characteristics of granular media is crucial for predicting how soils behave under structural loads. The test results, which illustrate the crushing force and deformation behavior of glass granules, provide a foundation for developing or calibrating models that simulate granular soil behavior, especially in the scenarios involving high compressive stresses or where particle breakage plays a role. The similarity between the stress–deformation responses of the glass granules and natural sand within practical stress ranges suggests that glass granules can be used to study load distribution, force chains, and strain localization in granular soils, which are critical aspects for engineering applications like retaining wall stability.

Moreover, because the glass granules are transparent, they allow the use of elasto-optical techniques (such as photoelasticity) to visualize the force chains and stress distribution within the material under a load. This is particularly useful in model studies of retaining walls or foundation loads, where understanding the internal force pathways and stress accumulation is essential for assessing the stability and failure mechanisms. The visualization of these “force chains” helps in identifying the critical load-bearing zones, which are directly relevant for soil mechanics applications when designing stable and efficient geotechnical structures.

The test results on the glass granules provide a reliable basis for the model simulations in soil mechanics, where they can effectively represent the behavior of natural soils. These findings contribute to refining the computational models and improving the experimental approaches in geotechnical engineering.

## Data Availability

The original contributions presented in the study are included in the article, further inquiries can be directed to the corresponding author.
